# How Similar Are the Mice to Men? Between-Species Comparison of Left Ventricular Mechanics Using Strain Imaging

**DOI:** 10.1371/journal.pone.0040061

**Published:** 2012-06-29

**Authors:** Kenya Kusunose, Marc S. Penn, Youhua Zhang, Yuanna Cheng, James D. Thomas, Thomas H. Marwick, Zoran B. Popović

**Affiliations:** 1 Department of Cardiovascular Medicine, Cleveland Clinic, Cleveland, Ohio, United States of America; 2 Department of Molecular Cardiology, Cleveland Clinic, Cleveland, Ohio, United States of America; University Hospital of Würzburg, Germany

## Abstract

**Background:**

While mammalian heart size maintains constant proportion to whole body size, scaling of left ventricular (LV) function parameters shows a more complex scaling pattern. We used 2-D speckle tracking strain imaging to determine whether LV myocardial strains and strain rates scale to heart size.

**Methods:**

We studied 18 mice, 15 rats, 6 rabbits, 12 dogs and 20 human volunteers by 2-D echocardiography. Relationship between longitudinal or circumferential strains/strain rates (S_Long_/SR_Long_, S_Circ_/SR_Circ_), and LV end-diastolic volume (EDV) or mass were assessed by the allometric (power-law) equation Y = kM^β^.

**Results:**

Mean LV mass in individual species varied from 0.038 to 134 g, LV EDV varied from 0.015 to 102 ml, while RR interval varied from 81 to 1090 ms. While S_Long_ increased with increasing LV EDV or mass (β values 0.047±0.006 and 0.051±0.005, p<0.0001 vs. 0 for both) S_Circ_ was unchanged (p = NS for both LV EDV or mass). Systolic and diastolic SR_Long_ and SR_Circ_ showed inverse correlations to LV EDV or mass (p<0.0001 vs. 0 for all comparisons). The ratio between S_Long_ and S_Circ_ increased with increasing values of LV EDV or mass (β values 0.039±0.010 and 0.040±0.011, p>0.0003 for both).

**Conclusions:**

While S_Circ_ is unchanged, S_Long_ increases with increasing heart size, indicating that large mammals rely more on long axis contribution to systolic function. SR_Long_ and SR_Circ_, both diastolic and systolic, show an expected decrease with increasing heart size.

## Introduction

The structure of mammalian hearts is grossly invariant between the species. Heart size maintains constant proportion to whole body size, while left ventricular (LV) myocardial histology shows a stable pattern of distribution of fibers that are oriented obliquely in subendo- and subepicardium, and circumferentially in mid-myocardium in all species. [1]–[2]. In contrast, myocardial function parameters show complex scaling relationships to body size. These relationships are driven by the fact that smaller animals consume more energy per body weight. [3] Because of this, scaling of functional indices, such as heart rate intervals, myocardial long axis velocities, or intraventricular pressure differences, can be expected. While the link between organ function and body (or organ) size can be modeled by several nonlinear mathematical relationships, allometric (power law) equation is most widely used as it is parsimonious (i.e. with a smallest number of parameters), and because the value of scaling coefficient has an intrinsic significance. [4].

What is less understood is the impact of heart size on LV deformational mechanics, namely myocardial strain and strain rate. Normal myocardial strains quantify myocardial deformation occurring in the direction of the three principal axis of the heart, with longitudinal strain (S_long_) reflecting long axis, and circumferential (S_circ_) and radial (S_rad_) strains reflecting corresponding components of short-axis deformation. Liu et al. have shown that S_circ_ and S_rad_ are decreased in mice and rats. [5] We have previously shown that ratio of long axis versus short axis displacement (which grossly represents ratio of S_long_ to S_circ_) decreases with decreasing heart size. [6] However, studies that quantitate the relationship between various strain components and body/organ size are lacking.

Systolic and diastolic strain rates, in turn, represent the rate of deformation. Strain rates are purportedly linked to contraction and relaxation at a more basic level. [7]–[8] Scaling of strain rates should be dependent on the interaction between scaling of myocardial velocities and left ventricular dimensions. Several papers, using both tissue Doppler and speckle tracking imaging, showed considerably higher strain rate in small rodents [9], [10], [11] and in smaller individuals within the single species [12], [13], [14]. However, no systematic assessment of the impact of heart size on strain rates has ever been performed.

In this paper we use echocardiography to assess three normal components of strains and corresponding strain rates in five mammalian species that differ in their body size by more than three orders of magnitude. The aim of this study was to determine the presence and magnitude of the impact of heart size on strains and strain rates using an allometric scaling model. Our research hypotheses were that 1) normal myocardial strains show a small but significant decrease with decreasing heart size [5] (See Appendix S1 and Figure S1 for further details), 2) strain rates show a large increase with decreasing heart size (see Appendix S1), and 3) S_long_ to S_circ_ scale differently to changing heart size. [6].

## Methods

### Study Population

Out of our database of 117 healthy volunteers free of any known cardiovascular disease, with normal physical examination, normal electrocardiogram, and not taking any cardio-active medication, we randomly selected 20 subjects whose age was between 21 and 45 years.

The studies in human subjects were performed in accordance with the Declaration of Helsinki. The Institutional Review Board of the Cleveland Clinic approved that study, and all participants gave written informed consent. The animal data were collected during echocardiographic evaluation of control (normal) animals used in several different studies in the period of 2003–2011. The data collection in each of these studies was prospective and specified by the respective study protocols. Importantly, the evaluation of the reported echocardiographic data was performed in each study and animal by using compatible equipment and techniques. All the animal experiments reported in this article were approved by the Institutional Animal Care and Use Committee of Cleveland Clinic and were in compliance with the National Institutes of Health Guide for the Care and Use of Laboratory Animals.

**Table 1:** Basic morphometric and echocardiography data of different species.

	n	Body weight(kg)	RR interval(ms)	ET(ms)	LV D/L ratio	EDV(mL)	ESV(mL)	EF	LV mass(g)
**Mouse**	18	0.033±0.006	92.5±11.1	39±6	0.49±0.08	0.032±0.017	0.009±0.004	0.68±0.06	0.068±0.02
**Rat**	15	0.264±0.025	143.2±17.1	63±8	0.52±0.10	0.255±0.09	0.076±0.028	0.69±0.07	0.728±0.176
**Rabbit**	6	3.01±0.39	296.8±36.1	125±17	0.66±0.11	2.9±0.8	1.1±0.5	0.61±0.08	3.6±0.5
**Dog**	12	26.3±5.9	501.3±38.8	157±12	0.65±0.10	57.4±5.9	22±6.9	0.62±0.06	86.1±19
**Human**	20	61.7±9.8	891.1±116.1	297±28	0.55±0.05	83.5±13	32.9±8.5	0.61±0.07	100.3±23.6

Abbreviations: ET: ejection time; D/L ratio: diameter/length ratio (diastole); EDV: end diastolic volume; ESV: end systolic volume; EF: ejection fraction; LV: left ventricular.

**Figure 1:**
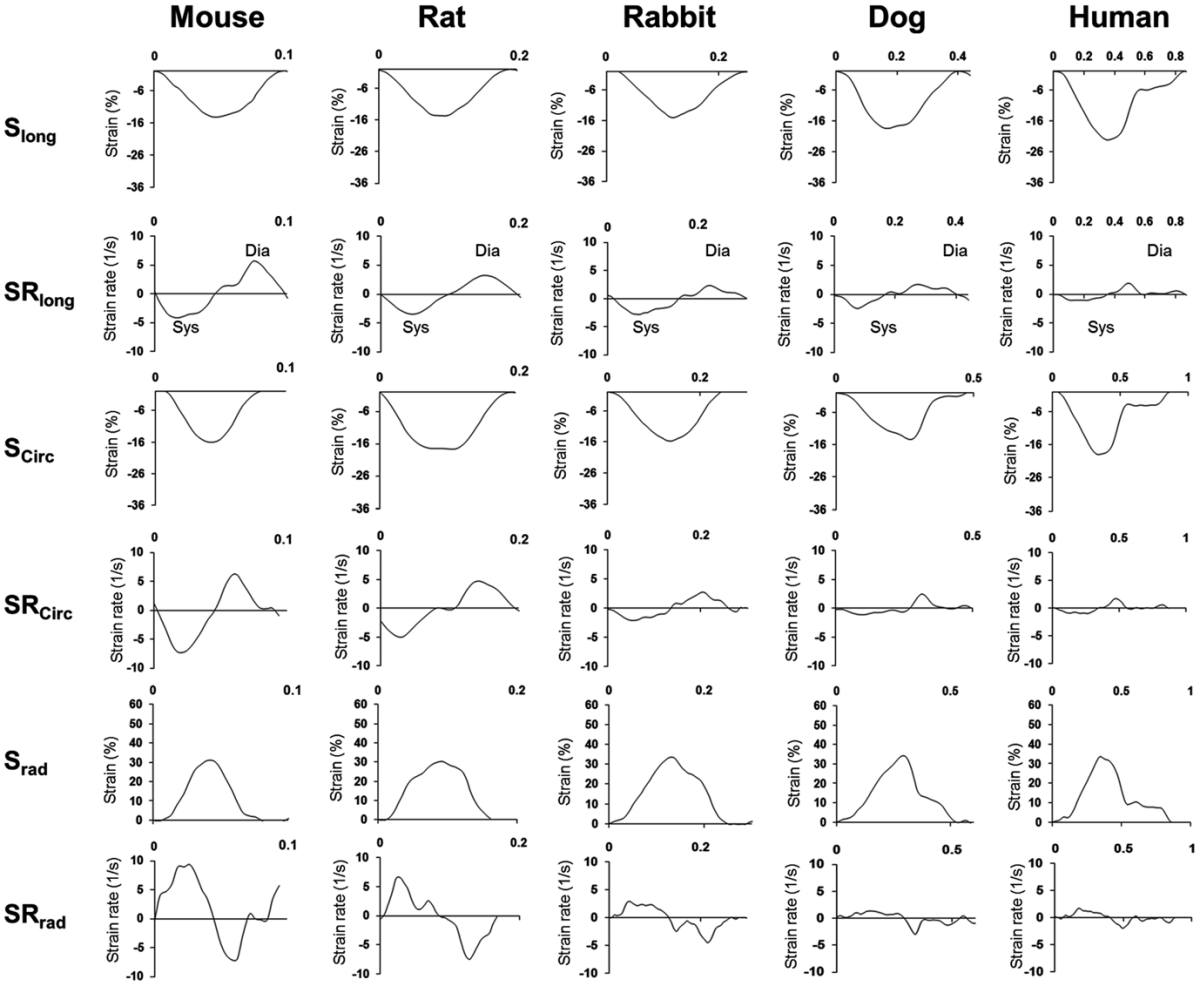
Examples of longitudinal, circumferential and radial myocardial strain and strain rate profiles in different species studied.

### Procedures

Subjects were studied in left lateral decubitus position. Due to the specificity of the animals’ behavior, the procedures differed slightly among the species. Twelve mongrel dogs were trained to lie down calmly for echocardiography. Six white New Zealand rabbits were lightly sedated with 35 mg/kg ketamine administered intramuscularly, with the handler keeping the head covered with soft cloth in a dark room. [15] Fifteen Lewis rats were sedated with 85 mg/kg of ketamine intraperitoneally [16] After ketamine administration, both rats and rabbits responded to transducer application to the chest wall, and they had to be gently restrained by the handler. Finally, eighteen C57/BL6 mice were assessed in a conscious state, with each one of them having at least one previous echocardiography session with the same handler. All animals were of adult age.

### Data Collection

Echocardiography was performed using Vivid 7 echocardiography machine (GE Medical, Milwaukee, WI, USA). M-mode echocardiography, two dimensional echocardiography, and two-dimensional color tissue Doppler echocardiography data were collected using a dual harmonic 1.7/3.4 MHz or 2.0/4.2 MHz sector transducer (humans, dogs), 6 MHz or 11.5 MHz pediatric sector transducer (rabbits, rats) and 14 MHz epicardial linear transducer (mice). The minimal frame rates acquired during standard two-dimensional echocardiography in humans, dogs, rabbits, rats and mice were 30, 50, 70, 90 and 160 frames s−1, respectively. The minimal frame rates acquired during color tissue Doppler two-dimensional echocardiography in humans, dogs, rabbits, rats and mice were 125, 125, 189, 200 and 208 frames s−1, respectively. Data were digitized in a proprietary format for further analysis. Long axis views were obtained from parasternal window in mice, rats and rabbits, and from apical window in dogs and human.

**Figure 2:**
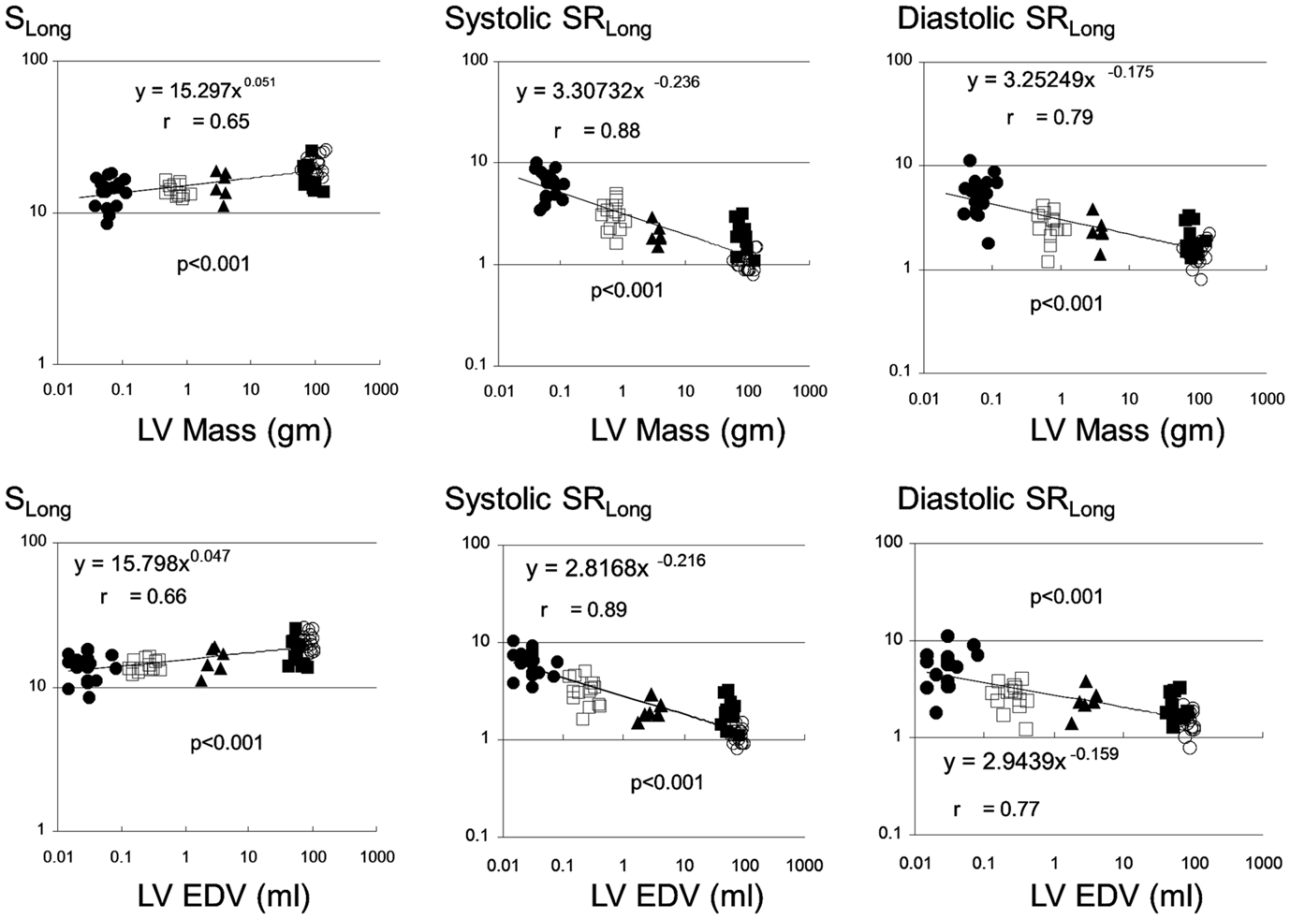
Scaling of longitudinal strain (S_long_) and peak systolic and early diastolic strain rates (SR_long_) to left ventricular end-diastolic volume and mass. Filled circles, empty squares, filled triangles, filled squares, and empty circles represent mice, rats, rabbits, dogs, and humans, respectively. EDV: end-diastolic volume; LV: left ventricle.

### Data Analysis

Data were analyzed using Echopac PC (GE Medical Systems, Milwaukee, Wi). LV ejection time was measured directly from the digital pulsed-wave Doppler tracing of the LV outflow tract with the sweep speed set to 200 mms^−1^. LV end-diastolic and end-systolic volumes were measured from the long-axis view by the single-plane Simpson equation. LV mass was calculated from two-dimensional echocardiography data by the bullet equation:

LV mass (g) = 1.05(5/6)[Aepi(L +√Aepi/π−√Aendo/π) − AendoL]

where 1.05 is the specific gravity of muscle, Aepi and Aendo are the epi- and endocardial parasternal short-axis areas, respectively, and L is the parasternal long-axis LV length. LV mass was calculated using both end-systolic and end-diastolic data, and the two estimates were then averaged. Previous studies have validated LV mass measurement based on two-dimensional echocardiography in mice [17], rabbits [18], dogs [19] and humans [20]. Additionally, the validation of LV mass estimation by the bullet equation in mice, rats, dogs and humans from our laboratory was already published. [6].

**Figure 3:**
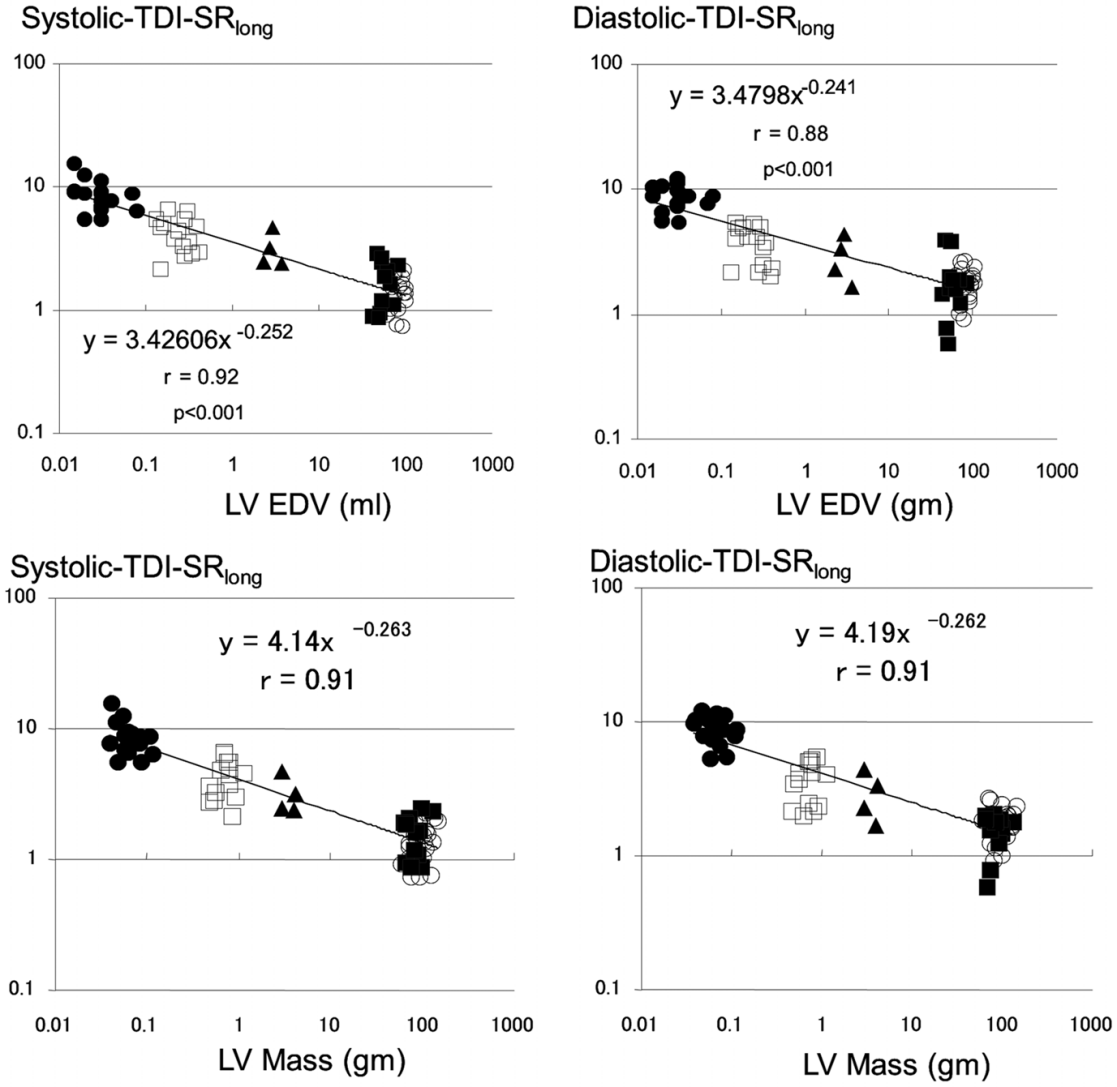
Scaling of longitudinal peak systolic and early diastolic strain rates obtained by tissue Doppler (TDI-SR_long_) to left ventricular end-diastolic volume and mass. Legend as for Figure 2.

#### Speckle tracking imaging

We used a speckle-tracking algorithm incorporated into Echopac PC PC (GE Medical Systems, Milwaukee, Wi). The speckle-tracking analysis was performed by the same trained observer. The region of interest was overlaid across a cross section of the ventricular silhouette at the image corresponding to the minimal endocardial area. The software algorithm then automatically divided the LV long-axis view and LV short-axis view into six segments for speckle tracking throughout the cardiac cycle. The tracking quality was then visually inspected, and, if it was satisfactory for at least five segments, the tracing was accepted. In mice, since our experiments were done in conscious setting without the possibility of registering the electrocardiogram, we manually defined end- systole and end-diastole. [21] Segmental longitudinal strain (S_long_) and strain rate (SR_long_), circumferential strain (S_circ_) and strain rate (SR_circ_), and radial strain (S_rad_) and strain rate (SR_rad_) curves were then constructed and then averaged to obtain global segmental strain and strain rate curves. End-systolic strains were then obtained from the global strain curves, while peak systolic and diastolic strain rates were calculated from global strain rate curves. For the analysis of human, dog and rabbit data, at least three beats were measured. For small rodents, at least six beats (for rats) or nine beats (for mice) were averaged. The mean value was used for statistical analysis. Long – short axis strain ratio (L/C strain ratio) was calculated by dividing longitudinal strain by circumferential strain.

**Figure 4:**
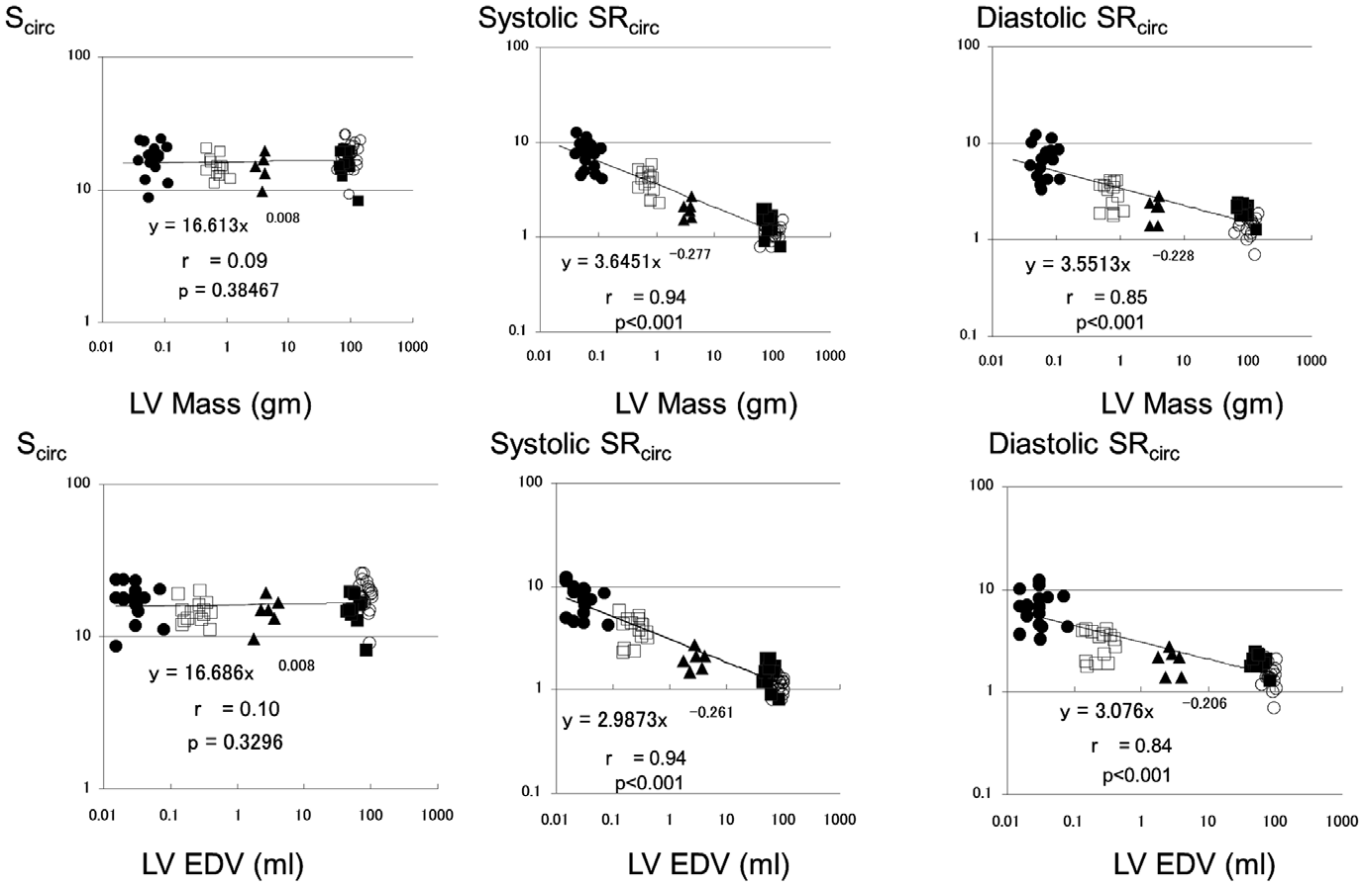
Scaling of circumferential strain (S_circ_) and corresponding peak systolic and early diastolic strain rates (SR_circ_ ) to left ventricular end-diastolic volume and mass. Legend as for Figure 2.

#### Tissue doppler imaging

Tissue Doppler Imaging, while carrying higher noise and larger operator variability, has a higher sampling rate and therefore potentially better detection of short-duration, high frequency peaks. [22] Therefore, in order to check for possible underestimation of strain rates, we also assessed Tissue Doppler imaging data obtained in a 4 chamber view and analyzed using EchoPAC PC (GE Medical Systems, Milwaukee, Wi). [10], [11] Diastolic and systolic longitudinal strain rates (TDI-SR_long_) were measured in mid interventricular septum using a strain rate length/region of interest of 0.6 mm/0.6 mm, 1.5 mm/1 mm, 3 mm/3 mm, 12 mm/6 mm and 15 mm/6 mm in mice, [23] rats, rabbits, dogs and humans, [24] respectively. The temporal smoothing filters were turned off for all measurements. The values obtained in 6 consecutive (mice and rats) or 3 consecutive (rabbits, dogs, and humans) cardiac cycles were averaged.

**Figure 5:**
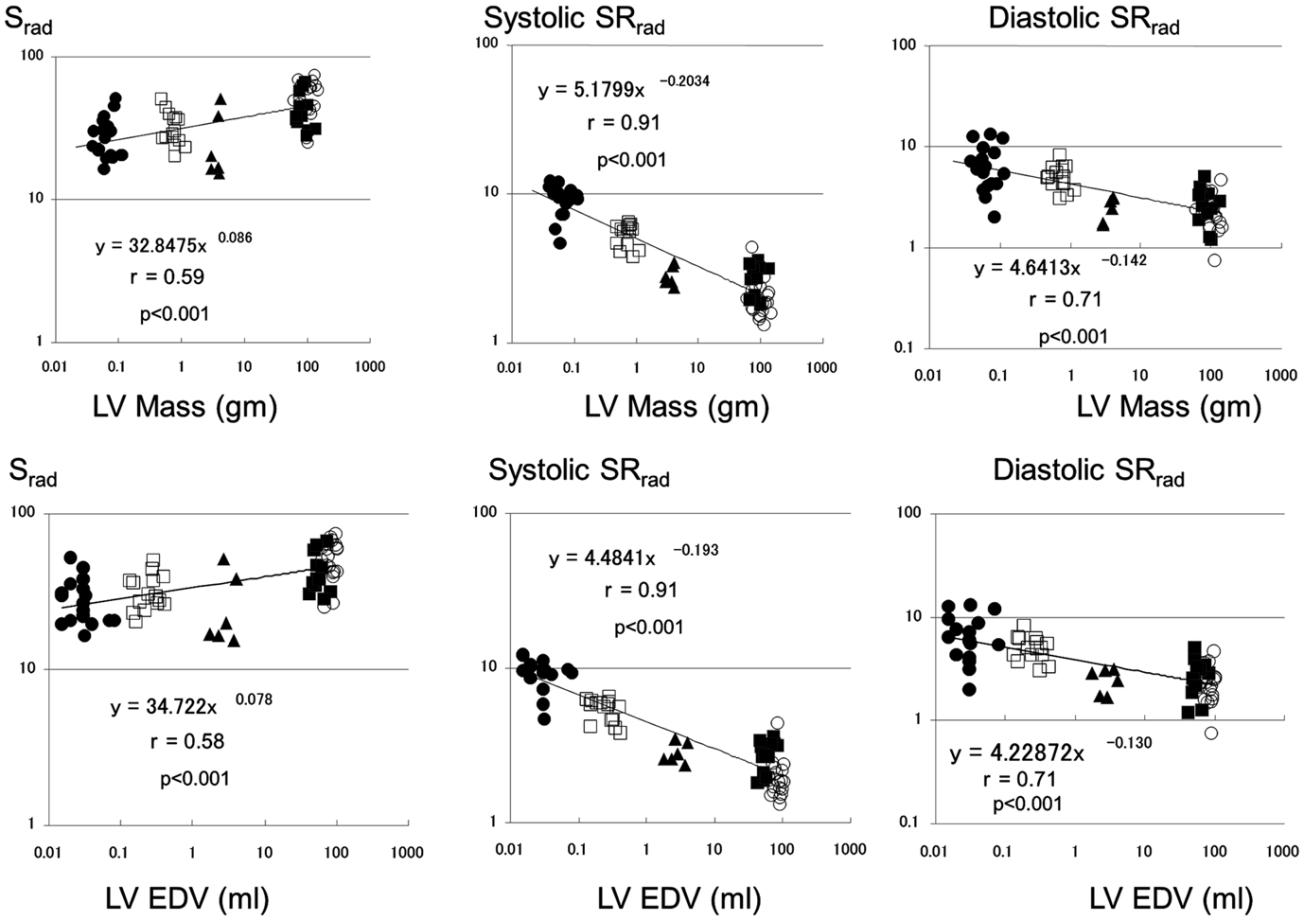
Scaling of S_rad_ and corresponding peak systolic and early diastolic strain rates (SR_rad_) to left ventricular end-diastolic volume and mass. Legend as for Figure 2.

**Figure 6:**
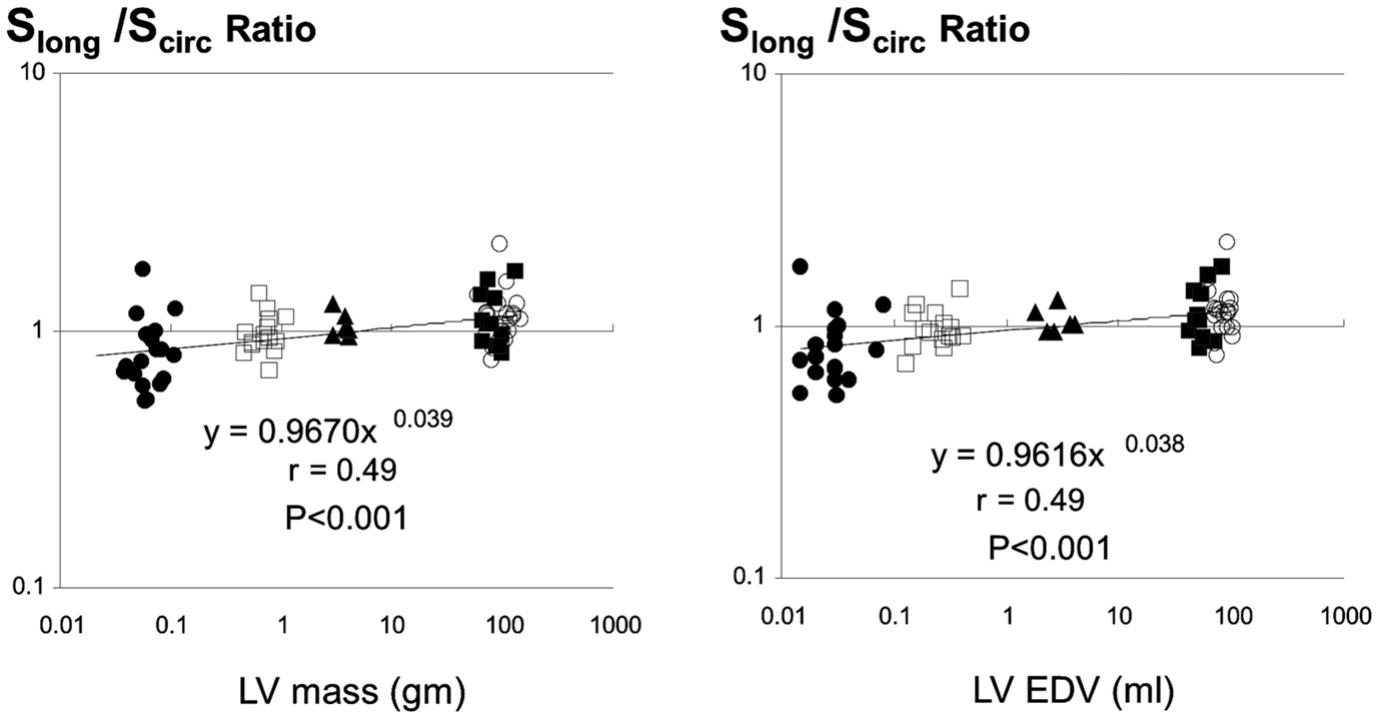
Scaling of long to short axis strain ratio (S_long_/S_circ_ Ratio) to left ventricular end-diastolic volume and mass.

### Quantifying Observer Variability

To estimate intraobserver variability, we used data obtained in 2 mice, 2 rats, 2 rabbits, 2 dogs, and 2 humans. The same observer applied speckle tracking software twice to the same cardiac cycle. Data are presented as means of the absolute and relative differences between measurements and by the correlation coefficient (r).

### Statistical Analysis

We used proposed power-law function to predict the magnitude of the impact of LV size on strain and strain rate [25]:
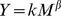
(1)where M is LV heart size parameter (i.e. LV EDV or LV mass), Y is a dependent variable (i.e. strain, strain rate), k is constant, and β is the power of the scaling exponent. To fit the data into the power function (1), we used a nonlinear regression method with Levenberg-Marquardt algorithm (SPSS 10.0, SPSS, Inc., Chicago, IL, USA). Confidence intervals (95%) for exponent β were calculated using an asymptotic error of estimate. A t test statistics was used to compare the values of exponent β compared to zero and obtained by different methods. [26]. Data are presented as mean ± SD or as exponent β ±standard error of estimate. A p value <0.05 was considered significant.

**Table 2:** Intra-inter observer variability of reconstructed strain data in the different species.

	LongitudinalStrain(%)	Systolic LongitudinalStrain Rate(^−s^)	Diastolic LongitudinalStrain Rate (^−s^)
Mean	23.1±13.5	3.6±2.6	3.4±1.8
Intraobserver variability			
Mean absolute difference	1.84±1.27	0.45±0.29	0.49±0.24
Mean relative difference (%)	8.4±5.2	12.5±7.4	13.3±7.2
Correlation	0.99	0.99	0.98

## Results

Basic morphometric and echocardiography parameters of different species are presented in Table 1. In our subjects, LV mass varied from 0.038 to 134 g, LV EDV varied from 0.015 to 102 ml, while RR interval varied from 81 to 1090 ms. Thus, body weight and heart size varied by up to four orders of magnitude among the subjects. Figure 1. represents characteristic individual myocardial strain and strain rate profiles in different species.

### Scaling of Long-axis Function

S_long_ showed a small but significant increase with increasing LV EDV and LV mass, with power exponent of β_EDV_ 0.047±0.006, and β_LVmass_ 0.051±0.007, respectively (p<0.0001 vs. 0 for both). As expected, absolute strain rates decreased with increased heart size, with systolic SR_long_ scaling with exponent of β_EDV_ −0.216±0.019, and β_LVmass_ −0.236±0.020 (p<0.0001 vs. 0 for both), and diastolic SR_long_ scaling with exponent of β_EDV_ −0.159±0.020, and β_LVmass_ −0.175±0.021 (p<0.0001 vs. 0 for both) (Figure 2).

DTI-derived strain rates also showed inverse relationships with the heart size. Systolic-TDI-SR_long_ scaled to LV EDV and mass with exponent of β_EDV_ −0.252±0.021 and of β_LVmass_ −0.263±0.021 (p<0.0001 vs. 0 for both; p = NS when compared to corresponding exponents obtained for speckle tracking-derived SR), while diastolic-TDI-SR_long_ scaled with exponent of β_EDV_ −0.241±0.020 and of β_LVmass_ −0.262±0.020 (p<0.0001 vs. 0 for both; p<0.01 when compared to corresponding exponents obtained for speckle tracking-derived SR) (Figure 3).

### Scaling of Short-axis Function

S_circ_ was not affected with heart size (β_EDV_ = 0.008±0.008, and β_LVmass_ = 0.008±0.009, p>0.1 vs. 0 for both). Again, systolic SR_circ_ decreased with increasing heart size with exponent of β_EDV_ −0.261±0.021, and β_LVmass_ −0.277±0.021 (p<0.0001 vs. 0 for both). Similarly, diastolic SR_circ_ scaled with exponent of β_EDV_ −0.206±0.023, and β_LVmass_ −0.228±0.023 (p<0.0001 vs. 0 for both) (Figure 4).

S_rad_ scaled with exponent of β_EDV_ 0.078±0.012, and β_LVmass_ 0.086±0.014 (p<0.0001 vs. 0 for both). Systolic SR_rad_ scaled with exponent of β_EDV_ −0.193±0.012, and β_LVmass_ −0.203±0.013 (p<0.0001 vs. 0 for both). Diastolic SR_rad_ scaled with exponent of β_EDV_ −0.130±0.018, and β_LVmass_ −0.142±0.020 (p<0.0001 vs. 0 for both) (Figure 5).

### Relationship between Long- and Short-axis Function

As a result, the longitudinal-to-circumferential strain ratio showed significant scaling exponents (β_EDV_ 0.038±0.009 and β_EDV_ 0.039±0.010, p = 0.0002 for both when compared with 0) (Figure 6). These results indicate that long- and short-axis strain scale differently.

### Relationship between Systolic and Diastolic Strain Rate

During data review, we noted that diastolic strain rates have consistently lower absolute values of power exponent. To elucidate this, we assessed the way the ratio between systolic and diastolic strain rates scales with LV EDV. All three ratios showed highly significant power exponent β (β for ratio between systolic and diastolic SR_long_ = −0.07±0.02, p<0.0001; β for ratio between systolic and diastolic SR_circ_ = −0.06±0.01, p<0.0001; β for ratio between systolic and diastolic SR_rad_ = −0.06±0.02, p<0.0001).

### Intra-observer Variability

Intra-observer variability data are shown in Table 2.

## Discussion

In this paper, we show that while S_Circ_ is unchanged, S_Long_ and S_rad_ increases with increasing heart size. Furthermore, there was a significant decrease of the ratio between S_Long_ and S_Circ_ with decreasing heart size, indicative that contribution of long axis function decreases with decreasing animal size. We also show that all three components of diastolic and systolic strain rates increased with the decreasing heart size while following an allometric (power law) function, with the power exponents similar to the ones *a priori* proposed by us.

From a practical viewpoint, our findings are relevant for the accurate translation of research from bench to bedside. Additionally they are important in the context of evolutionary biology, as we describe the way cardiac structures adopt to size constraints by changing the way they operate.

### Strain and Heart Size

Myocardial deformation is a complex phenomenon that can best be represented as a 3×3 tensor, with diagonal elements representing three normal strains (analyzed in this paper), and off-diagonal elements representing shear strains. LV myocardial deformation during systole leads to expulsion of blood, with this process most often quantified by ejection fraction, a measure of global systolic function. As ejection fraction is constant between the species. [5], [6] it follows that myocardial strains should be largely invariant between the species. Yet Liu et al. showed that anesthetized mice have markedly longitudinal shortening (by 10%) and S_circ_ lower, but by a lesser degree (by 6%), than conscious humans [5]. In contrast Bachner-Hinenzon et al. [27] showed that sedated rats have S_circ_ that is 2% lower, but S_long,_ that is similar to humans; of note, this study reported markedly lower human S_long_ than the one reported in the largest clinical multi-center S_long_ study of healthy controls [28]. Our data show similar S_circ_ between species but S_long_ that was 5% lower in mice than in humans. A possible discrepancy between these studies may be due to differences in methods and/or the use of anesthetics/sedatives [21]. On the other hand, our findings of a small but significant increase of S_long_/S_circ_ ratio with increasing animal size are in accordance with the previously shown increase of the ratio between long- and short-axis displacement with animal size increase, [6] and is supported by findings of Liu et al. that shows that body size has a stronger effect on long axis function. Finally, Decloedt et al. have shown that trotter horses [29], [30] compared to humans reported in our study and previously [28] [27] have higher S_long_ of −24.6% but similar S_circ_ of −19.7%, again consistent with increase in S_long_/S_circ_ ratio with increasing animal size.

### Strain Rates and Heart Size

We confirmed that strain rates scaled to the heart size with power exponents that were similar to the ones proposed in Appendix S1. The largest divergence from the predicted exponent was seen for diastolic and systolic SR_rad_, which could represent a true physiologic phenomenon, or may be due to difficulties in accurately measuring radial strain rate. As a further support for our findings, Decloedt et al. have shown that trotter horses have mean values of all strain rate components lower by almost one order of magnitude when compared to the ones we obtained in mice [29], [30].

It is interesting that for all three components of global strain rates, diastolic strain rates showed lower values of the power exponent. This would indicate that decreasing heart size leads to a larger increase in systolic, rather than diastolic strain rate. For example, in our data, the average systolic and diastolic SR_long_ by speckle tracking were 1.0^−s^ and 1.5^−s^ in humans, but 6.5^−s^ and 4.3^−s^ in mice. This issue is even more complex given relatively shorter diastolic filling time in small mammals. [16] While the cause of this finding it unknown, it may be linked to between-species differences in gene expression. [31], [32] Further studies are needed to elucidate if these findings carry evolutionary importance. [33].

### Limitations

Several confounding factors, such as differences in heart rate to frame rate ratio, impact of sedation, or intrinsic differences in heart geometry) could have influenced the results. However, recent report showed that speckle-tracking LV strain efficiently detects discrete alterations in mice despite being obtained from images acquired at the frame rate identical to one used in this study. [34] Also, mice, the smallest species studied, have been assessed without any sedation. Finally, LV geometry, quantified by the ratio between LV radius and length, while showing between-species variability, [5] appears to vary independently from the species size (see Table 2).

In this study LV mass of our human subjects was lower than reported in our previous studies, which decreased the range of LV mass studied. The reason for this was that 14 out of 20 of our subjects were women with relatively small body size. The average indexed LV mass was 61±12 gm/m^2^, i.e. within normal limits.

Speckle-tracking derived strains obtained by method used in this study were validated only in humans and dogs, [35] which makes accuracy of assessment of strain in small animals uncertain. However, we have shown that speckle-tracking derived strains derived by the current method are reflective of myocardial scar or fibrosis in rabbits, rats, and mice. [9], [15], [21] We are also showing here that high-frame tissue-Doppler derived strain rate of the mid-ventricular septum (which was validated in men [36] and in mice, [37] i.e. in species at the two extremes of body size) correlates well with the global speckle-tracking derived strain rate, adding to plausibility of our findings.

We have not assessed all strain components, and the significant impact of shear strains cannot be excluded. Also, strain rate measurements by speckle tracking echocardiography are less accurate than the strain measurements. Yet several authors showed that one can accurately measure strain rates in mice and rats, and that these measures correlate with relevant physiologic parameters. Furthermore, we tried to overcome this issue by averaging strain from multiple segments. Finally, the impact of sedation and the stress of performing echocardiography studies cannot be completely eliminated.

In conclusion, while S_Circ_ is unchanged, S_Long_ and S_rad_ increase with increasing heart size, indicating that large mammals rely more on long axis contribution to systolic function. While both diastolic and systolic SR_Long,_ SR_Circ_, and SR_Rad_ increase with decreasing heart size, the increase is more substantial for systolic SR. Further studies are needed to develop species normal ranges for subsequent intervention studies.

## Supporting Information

Figure S1 **Scaling of left ventricular (LV) length and systolic and diastolic mitral annulus (MA) long axis velocities to left ventricular mass derived from previous data** [**6**] **(Panels A and B); Predicted scaling of longitudinal systolic and diastolic strain rates (SR_long_) (Panels C and D); Scaling of longitudinal and circumferential strains (S_long_ and S_circ_) derived from data from Liu et al.** [**5**] **(Panel E).**
(TIF)

Appendix S1 **Study Background.**
(DOC)

## References

[1] Grimm AF, Katele KV, Lin HL (1976). Fiber bundle direction in the mammalian heart. An extension of the “nested shells” model.. Basic Res Cardiol.

[2] Costa KD, May-Newman K, Farr D, O’Dell WG, McCulloch AD (1997). Three-dimensional residual strain in midanterior canine left ventricle.. Am J Physiol.

[3] Prothero J (1979). Heart weight as a function of body weight in mammals.. Growth.

[4] Noujaim SF, Lucca E, Munoz V, Persaud D, Berenfeld O (2004). From mouse to whale: a universal scaling relation for the PR Interval of the electrocardiogram of mammals.. Circulation.

[5] Liu W, Ashford MW, Chen J, Watkins MP, Williams TA (2006). MR tagging demonstrates quantitative differences in regional ventricular wall motion in mice, rats, and men.. American Journal of Physiology - Heart and Circulatory Physiology.

[6] Popovic ZB, Sun JP, Yamada H, Drinko J, Mauer K (2005). Differences In Left Ventricular Long Axis Function From Mice To Humans Follow Allometric Scaling To Ventricular Size.. J Physiol.

[7] Armstrong G, Pasquet A, Fukamachi K, Cardon L, Olstad B (2000). Use of peak systolic strain as an index of regional left ventricular function: Comparison with tissue Doppler velocity during dobutamine stress and myocardial ischemia.. Journal of the American Society of Echocardiography.

[8] Greenberg NL, Firstenberg MS, Castro PL, Main M, Travaglini A (2002). Doppler-derived myocardial systolic strain rate is a strong index of left ventricular contractility.. Circulation.

[9] Popovic ZB, Benejam C, Bian J, Mal N, Drinko J (2007). Speckle-tracking echocardiography correctly identifies segmental left ventricular dysfunction induced by scarring in a rat model of myocardial infarction.. Am J Physiol Heart Circ Physiol.

[10] Derumeaux G, Mulder P, Richard V, Chagraoui A, Nafeh C (2002). Tissue Doppler imaging differentiates physiological from pathological pressure-overload left ventricular hypertrophy in rats.. Circulation.

[11] Thibault H, Gomez L, Bergerot C, Augeul L, Scherrer-Crosbie M (2011). Strain-Rate Imaging Predicts the Attenuation of Left Ventricular Remodeling Induced by Ischemic Postconditioning After Myocardial Infarction in Mice.. Circulation-Cardiovascular Imaging.

[12] Oxborough D, Batterham AM, Shave R, Artis N, Birch KM (2009). Interpretation of two-dimensional and tissue Doppler-derived strain () and strain rate data: is there a need to normalize for individual variability in left ventricular morphology?. European Journal of Echocardiography.

[13] Rosner A, Bijnens B, Hansen M, How OJ, Aarsaether E (2008). Left ventricular size determines tissue Doppler-derived longitudinal strain and strain rate.. European Journal of Echocardiography.

[14] Kuznetsova T, Herbots L, Richart T, D’Hooge J, Thijs L (2008). Left ventricular strain and strain rate in a general population.. European Heart Journal.

[15] Wang YT, Popović ZB, Efimov IR, Cheng Y Longitudinal Study of Cardiac Remodelling in Rabbits Following Infarction.. Canadian Journal of Cardiology.

[16] Popovic ZB, Richards KE, Greenberg NL, Rovner A, Drinko J (2006). Scaling of diastolic intraventricular pressure gradients is related to filling time duration.. American Journal of Physiology - Heart and Circulatory Physiology.

[17] Collins KA, Korcarz CE, Shroff SG, Bednarz JE, Fentzke RC (2001). Accuracy of echocardiographic estimates of left ventricular mass in mice.. American journal of physiology Heart and circulatory physiology.

[18] Plehn JF, Foster E, Grice WN, Huntington-Coats M, Apstein CS (1993). Echocardiographic assessment of LV mass in rabbits: models of pressure and volume overload hypertrophy.. Am J Physiol.

[19] Wyatt HL, Heng MK, Meerbaum S, Hestenes JD, Cobo JM (1979). Cross-sectional echocardiography. I. Analysis of mathematic models for quantifying mass of the left ventricle in dogs.. Circulation.

[20] Reichek N, Helak J, Plappert T, Sutton MS, Weber KT (1983). Anatomic validation of left ventricular mass estimates from clinical two-dimensional echocardiography: initial results.. Circulation.

[21] Peng Y, Popovic ZB, Sopko N, Drinko J, Zhang Z (2009). Speckle tracking echocardiography in the assessment of mouse models of cardiac dysfunction.. American Journal of Physiology - Heart and Circulatory Physiology.

[22] Kukulski T, Voigt JU, Wilkenshoff UM, Strotmann JM, Wranne B (2000). A comparison of regional myocardial velocity information derived by pulsed and color Doppler techniques: An in vitro and in vivo study.. Echocardiography-a Journal of Cardiovascular Ultrasound and Allied Techniques.

[23] Popovic ZB, Richards KE, Greenberg NL, Rovner A, Drinko J (2006). Scaling of diastolic intraventricular pressure gradients is related to filling time duration.. American journal of physiology Heart and circulatory physiology.

[24] Sun JP, Popovic ZB, Greenberg NL, Xu XF, Asher CR (2004). Noninvasive quantification of regional myocardial function using Doppler-derived velocity, displacement, strain rate, and strain in healthy volunteers: effects of aging.. J Am Soc Echocardiogr.

[25] Zar JH (1984). Biostatistical Analysis.. Englewood Cliffs: Prentice-Hall, Inc.

[26] Dawson-Saunders B, Trapp RG (1990). Basic and clinical biostatistics. East Norwalk: Appleton&Lange.. 284 p.

[27] Bachner-Hinenzon N, Ertracht O, Leitman M, Vered Z, Shimoni S (2010). Layer-specific strain analysis by speckle tracking echocardiography reveals differences in left ventricular function between rats and humans.. American journal of physiology Heart and circulatory physiology.

[28] Marwick TH, Leano RL, Brown J, Sun JP, Hoffmann R (2009). Myocardial strain measurement with 2-dimensional speckle-tracking echocardiography: definition of normal range.. JACC Cardiovasc Imaging.

[29] Decloedt A, Verheyen T, Sys S, De Clercq D, van Loon G (2011). Quantification of left ventricular longitudinal strain, strain rate, velocity, and displacement in healthy horses by 2-dimensional speckle tracking.. J Vet Intern Med.

[30] Decloedt A, Verheyen T, Sys S, De Clercq D, van Loon G (2012). Two-dimensional speckle tracking for quantification of left ventricular circumferential and radial wall motion in horses.. Equine Vet J.

[31] Mercadier JJ, Lompre AM, Wisnewsky C, Samuel JL, Bercovici J (1981). Myosin Isoenzymic Changes in Several Models of Rat Cardiac-Hypertrophy.. Circulation Research.

[32] Schwartz K, Lecarpentier Y, Martin JL, Lompre AM, Mercadier JJ (1981). Myosin Isoenzymic Distribution Correlates with Speed of Myocardial-Contraction.. Journal of Molecular and Cellular Cardiology.

[33] Bers DM (2000). Calcium fluxes involved in control of cardiac myocyte contraction.. Circulation Research.

[34] Bauer M, Cheng S, Jain M, Ngoy S, Theodoropoulos C (2011). Echocardiographic Speckle-Tracking Based Strain Imaging for Rapid Cardiovascular Phenotyping in Mice.. Circulation Research.

[35] Amundsen BH, Helle-Valle T, Edvardsen T, Torp H, Crosby J (2006). Noninvasive myocardial strain measurement by speckle tracking echocardiography: validation against sonomicrometry and tagged magnetic resonance imaging.. J Am Coll Cardiol.

[36] Cho GY, Chan J, Leano R, Strudwick M, Marwick TH (2006). Comparison of two-dimensional speckle and tissue velocity based strain and validation with harmonic phase magnetic resonance imaging.. Am J Cardiol.

[37] Sebag IA, Handschumacher MD, Ichinose F, Morgan JG, Hataishi R (2005). Quantitative assessment of regional myocardial function in mice by tissue Doppler imaging: comparison with hemodynamics and sonomicrometry.. Circulation.

