# Supplementary figures and images for: How Similar Are the Mice to Men? Between-Species Comparison of Left Ventricular Mechanics Using Strain Imaging
Source: PLoS One. 2012 Jun 29;7(6):e40061. doi: 10.1371/journal.pone.0040061 (PMC3386935; doi:10.1371/journal.pone.0040061)

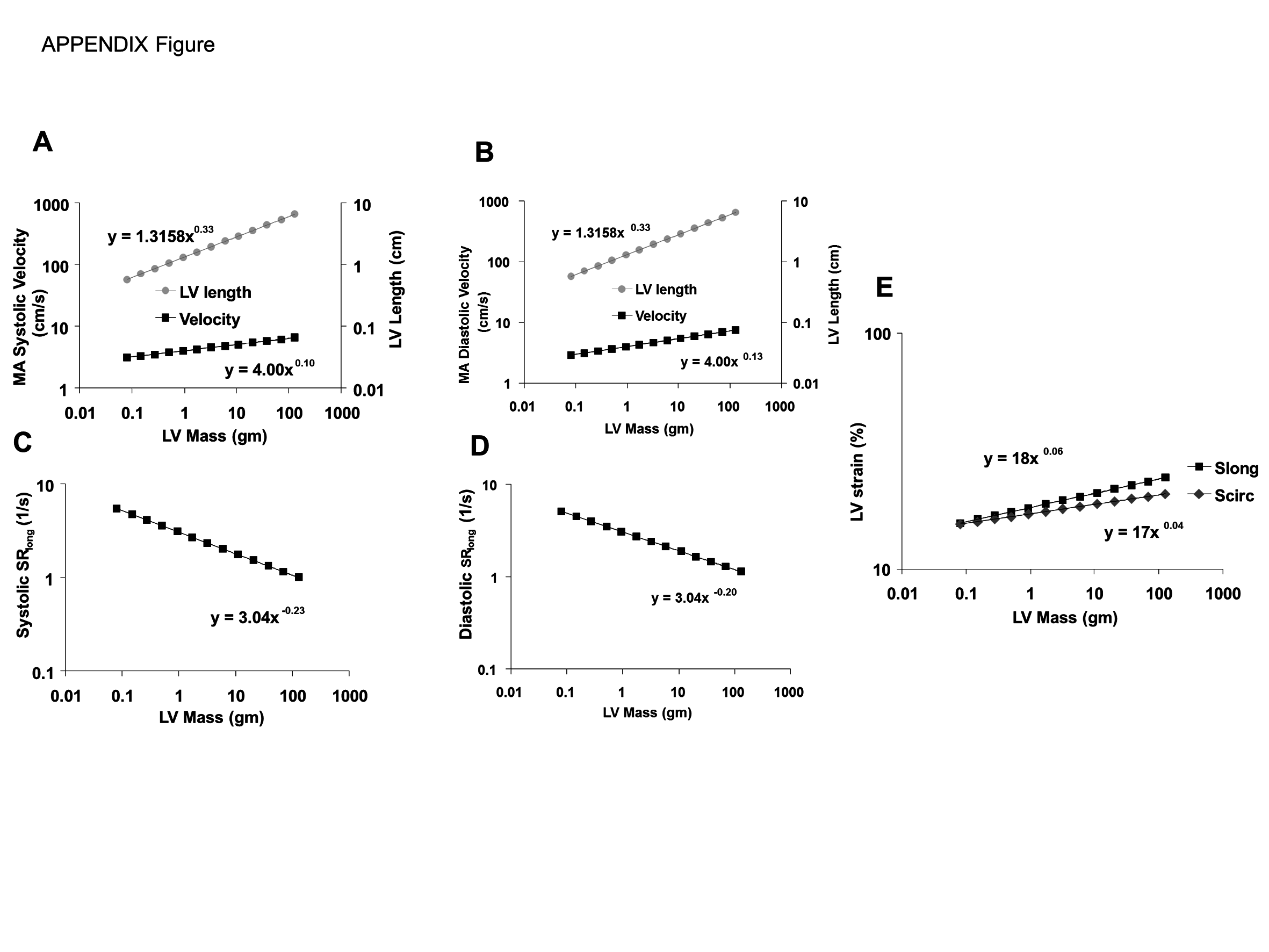

Supplement: Figure S1 — Scaling of left ventricular (LV) length and systolic and diastolic mitral annulus (MA) long axis velocities to left ventricular mass derived from previous data [6] (Panels A and B); Predicted scaling of longitudinal systolic and diastolic strain rates (SRlong) (Panels C and D); Scaling of longitudinal and circumferential strains (Slong and Scirc) derived from data from Liu et al. [5] (Panel E). (TIF) [file pone.0040061.s001.tif]
