# Supplementary material for: How Similar Are the Mice to Men? Between-Species Comparison of Left Ventricular Mechanics Using Strain Imaging
Source: PLoS One. 2012 Jun 29;7(6):e40061. doi: 10.1371/journal.pone.0040061 (PMC3386935; doi:10.1371/journal.pone.0040061)
Supplement: Appendix S1 — Study Background. (DOC) [file pone.0040061.s002.doc]

**How Similar Are the Mice to Men? Between-species Comparison of Left Ventricular Mechanics Using Strain Imaging**

Kenya Kusunose, MD, ^1^ Marc S. Penn, MD, PhD, ^1^ Youhua Zhang, MD, ^2^ PhD,

Yuanna Cheng, MD, PhD, ^2^ James D. Thomas, ^1^ MD, Thomas H. Marwick, MD, PhD, ^1^ and

Zoran B. Popović MD, PhD^1^

**Appendix**

**Study Background**

We have previously shown that long axis (mitral annulus) velocity scales to the LV mass with exponents b for systolic and diastolic myocardial velocities of 0.10 and 0.13, respectively. Strain rate represents a velocity gradient (i.e. spatial derivative of velocity), and in the hypothetical *in vitro* model of an isolated muscle strip with fixed proximal end is equal to myocardial velocity of distal end divided by initial myocardial length. With the *in vivo* measurement of long axis (mitral annulus) velocities, the initial length can be approximated by LV length. Assuming that LV shape is invariant, LV length scales to the LV mass according to power function with exponent β = 0.33. Given these initial parameters, one can predict scaling of systolic and diastolic strain rates with exponent β of -0.23 and -0.20, respectively. (Supplemental Figure 1. A-D). Using the data of Liu et al. showing S_circ_ values of 21%, 19% and 15%, and long axis shortening (measure related to S_long_) respective values of 24%, 19% and 15% in humans, rats, and mice to fit into equation 1, we obtained scaling exponents β of 0.06 and 0.04 for S_long_ and S_circ_ , respectively (Supplemental Figure 1. E).

Supplemental Figure 1. Scaling of left ventricular (LV) length and systolic and diastolic mitral annulus (MA) long axis velocities to left ventricular mass derived from previous data[6] (Panels A and B); Predicted scaling of longitudinal systolic and diastolic strain rates (SR_long_) (Panels C and D); Scaling of longitudinal and circumferential strains (S_long_ and S_circ_) derived from data from Liu et al. [5] (Panel E).
